# Multidisciplinary Management of Malignant Phyllodes Tumours of the Breast: A Case-Based Illustration and Systematic Review

**DOI:** 10.3390/ijms27104376

**Published:** 2026-05-14

**Authors:** Greta Di Stefano, Graziella Marino, Alexios Thodas, Pasqualina Modano, Grazia Lazzari, Antonietta Montagna, Tommaso Fabrizio, Massimo Dante Di Somma, Giulia Anna Carmen Vita, Giuseppina Dinardo, Marzia Sichetti, Marisabel Mecca, Alessio Vagliasindi

**Affiliations:** 1 Abdominal Oncological Surgery Unit, Centro di Riferimento Oncologico della Basilicata (IRCCS-CROB), 85028 Rionero in Vulture, Italy; greta.distefano@crob.it (G.D.S.); alessio.vagliasindi@crob.it (A.V.); 2 Breast Cancer Unit, Centro di Riferimento Oncologico della Basilicata (IRCCS-CROB), 85028 Rionero in Vulture, Italy; graziella.marino@crob.it (G.M.);; 3 Emergency and Palliative Care Unit, Centro di Riferimento Oncologico della Basilicata (IRCCS-CROB), 85028 Rionero in Vulture, Italy; 4 Radiation Oncology Unit, Centro di Riferimento Oncologico della Basilicata (IRCCS-CROB), 85028 Rionero in Vulture, Italy; 5 Plastic Surgery Unit, Centro di Riferimento Oncologico della Basilicata (IRCCS-CROB), 85028 Rionero in Vulture, Italy; 6 Anatomical Pathology Department, Centro di Riferimento Oncologico della Basilicata (IRCCS-CROB), 85028 Rionero in Vulture, Italy; 7 Diagnostic and Imaging Department, Centro di Riferimento Oncologico della Basilicata (IRCCS-CROB), 85028 Rionero in Vulture, Italy; 8 Laboratory of Preclinical and Translational Research, Centro di Riferimento Oncologico della Basilicata (IRCCS-CROB), 85028 Rionero in Vulture, Italy

**Keywords:** phyllodes tumours, breast cancer, surgical margins, radiotherapy outcomes, systematic review, molecular markers, multidisciplinary management

## Abstract

Phyllodes tumours (PTs) of the breast are rare fibroepithelial neoplasms with potentially aggressive behaviour, characterised by rapid growth, a significant risk of local recurrence, and occasional metastatic spread. Optimal management remains controversial, particularly regarding surgical margins, adjuvant radiotherapy, and the relevance of molecular markers in predicting tumour behaviour. A PRISMA 2020-guided qualitative systematic review was conducted of studies published between January 2000 and December 2024 in PubMed/MEDLINE, Scopus, and Web of Science. Eligible studies included malignant PTs of the breast and addressed at least one of the following domains: molecular pathology, surgical margins and local recurrence, adjuvant radiotherapy, or predictors of recurrence and metastasis. A clinical case of malignant PT treated at our institution is presented as an illustrative study. Thirty-four studies met the inclusion criteria. Evidence suggests that margin status, stromal proliferative activity, and selected molecular markers influence recurrence risk. Several retrospective studies suggest that adjuvant radiotherapy may improve local control in selected high-risk malignant PTs, although the evidence remains heterogeneous, retrospective, and potentially affected by treatment-selection bias, and no consistent survival benefit has been demonstrated. Molecular alterations, including *MED12* mutations, *TERT* promoter mutations, *TP53* alterations, and increased Ki-67 expression, have been associated with tumour progression and aggressive behaviour. A 44-year-old woman presented with a 2.4 cm left breast mass on radiological examination. Lumpectomy revealed a malignant PT with stromal hypercellularity, nuclear atypia, and a mitotic index of 20/10 HPF with close margins. Immunohistochemistry showed positivity for CD99, Bcl-2, and CD34 with a Ki-67 proliferation index of 20%. The patient underwent wide local re-excision followed by adjuvant radiotherapy (60 Gy), and at 24-month follow-up, the patient remained disease-free. Evidence synthesis highlights the importance of complete surgical excision, multidisciplinary management, and consideration of adjuvant radiotherapy in selected malignant PTs. Emerging molecular profiling may contribute to improved biological understanding and future risk stratification of malignant PTs, although its routine clinical utility remains to be validated in prospective studies.

## 1. Introduction

Phyllodes tumours (PTs) of the breast are uncommon tumours accounting for 0.3 to 1% of all breast tumours and 2.5% of all fibroepithelial breast tumours [1]. PTs are characterised by a double-layered epithelial component surrounded by a hypercellular mesenchymal stroma [2]. The average size of PTs is 4–5 cm; however, there are also giant PTs exceeding 10 cm in diameter, which account for approximately 20% of all PT cases [1].

The World Health Organization (WHO) classifies PTs as benign (60–75%), borderline (13–26%) or malignant (10–20%) [3,4] based on stromal cellularity, atypia, mitotic rate, stromal overgrowth, and tumour borders. PTs are more frequent in women between 35 and 55 years old [1,5,6,7], with a median age of 50 years for malignant tumours. The majority of PTs are localised (80%), while distant metastases have been reported in up to 22% of malignant PT cases [8]. However, axillary lymph node metastases are rare.

Surgical resection remains the standard treatment for all types of PTs and typically involves wide local excision without axillary dissection. Despite the rarity of malignant PTs, their management remains controversial because prospective trials are lacking, and treatment frequently depends on multidisciplinary evaluation.

The major unresolved clinical questions concern three interconnected domains. First, the optimal surgical margin is a matter of debate. Traditional recommendations have favoured wide excision with a margin of at least 1 cm, yet recent analyses suggest that the achievement of a histologically negative margin may be more important than the absolute width itself [9]. Second, the role of adjuvant radiotherapy (RT) is still debated. No randomised trial has demonstrated a survival benefit, but several retrospective cohorts indicate that RT may improve local control in selected high-risk situations [10]. Third, molecular pathology has rapidly evolved and now offers a biologically richer framework for understanding PT’s progression, differential diagnosis, and risk stratification [11]. In particular, the published literature supports a multistep biological model. *MED12* alterations are frequent early events shared with fibroadenomas, whereas *TERT*-promoter mutations, *TP53* abnormalities, copy-number alterations, and cell-cycle pathway disruptions become more prominent with increasing PT grade and metastatic behaviour [12,13,14,15].

Given the lack of prospective trials and the increasing availability of molecular evidence, a qualitative systematic review with an integrated case report can clarify the current state of knowledge. Therefore, the present manuscript is structured as a PRISMA-guided qualitative systematic review of malignant PTs of the breast, focused on three clinically relevant themes: (i) molecular markers and genomic alterations; (ii) surgical margins and recurrence; and (iii) adjuvant RT and treatment outcomes.

In this study, we describe a case illustration of a 44-year-old woman with a malignant breast PT, who underwent surgery and therapy at the Breast Surgery Unit of the Centro di Riferimento Oncologico della Basilicata (IRCCS-CROB) in Rionero in Vulture, southern Italy.

This case, reported in accordance with the Surgical CAse REport (SCARE) 2023 criteria [16], is retained as a case illustration to show how these evidence gaps affect real-world multidisciplinary decision-making.

## 2. Methods

### 2.1. Study Design

This study was conducted as a qualitative systematic review with an integrated illustrative case report following the Preferred Reporting Items for Systematic Reviews and Meta-Analyses (PRISMA 2020) guidelines for transparent reporting of the search, screening, eligibility criteria, and study selection process [17].

The objective was to evaluate the current clinicopathologic, molecular, and treatment-related evidence in malignant PTs of the breast. Therefore, the review addressed the following questions:

(1) Which molecular and immunohistochemical markers are associated with diagnosis, grading, recurrence, or aggressive behaviour in malignant PTs?
(2) What is the relationship between surgical margin status/width and local recurrence?
(3) What is the impact of adjuvant RT on local control, recurrence-free survival, and overall survival in malignant PTs?

### 2.2. Search Strategy

To strengthen the accompanying literature review, we performed a comprehensive search in specialised databases such as PubMed/MEDLINE, Scopus, and Web of Science Core Collection for studies published between January 2000 and December 2024.

The search string strategy combined the following keywords: (“phyllodes tumour” OR “phyllodes tumor” OR “malignant phyllodes” OR “breast phyllodes”) AND (“malignant” OR “borderline”) AND (“surgical margins” OR “margin status” OR “wide local excision” OR “mastectomy” OR “radiotherapy” OR “recurrence” OR “metastasis” OR “survival” OR “molecular markers” OR “MED12” OR “TERT promoter” OR “TP53” OR “Ki-67” OR “p53”).

Search strings were adapted for PubMed, Scopus, and Web of Science. The reference list of eligible studies and relevant reviews was manually screened to identify additional records. Studies were reviewed for population size, treatment modalities, recurrence, metastasis, and survival, and reference lists of relevant articles were also manually screened to identify additional eligible studies.

This review was not prospectively registered, and no formally registered protocol was available before study selection. However, to improve transparency, the review question, eligibility criteria, information sources, and search framework applied in the present revision are now fully reported in the Supplementary Materials, in accordance with the PRISMA 2020 reporting guidance.

### 2.3. Eligibility Criteria

Studies were included if they met the following criteria:

Inclusion criteria

- Population: Patients with histologically confirmed malignant PTs of the breast; mixed-grade PTs studies were eligible only if malignant cases were reported separately or could be extracted;
- Exposure/intervention: Molecular testing, immunohistochemistry, surgery with margin analysis, re-excision, adjuvant RT, or outcome predictors;
- Outcomes: Local recurrence, distant metastasis, disease-free survival, overall survival, pathological or molecular associations, or treatment-related outcomes;
- Study design: Eligible core evidence included retrospective or prospective cohort studies, registry-based analyses, multicentre studies, molecular or immunohistochemical studies with original data, and systematic reviews/meta-analyses when relevant to the predefined review questions. Narrative reviews, guidelines, expert commentaries, and classification papers were retained only as contextual/background references. Case reports and very small case series were not considered part of the core evidence synthesis and were used only as illustrative examples when relevant to rare presentations, reconstructive strategies, or multidisciplinary clinical decision-making;
- Language: English;
- Time window: From January 2000 to December 2024.

Exclusion criteria

- Studies involving non-breast PTs;
- Conference abstracts without full data;
- Narrative reviews without original data (for the main synthesis);
- Duplicate publications;
- Studies lacking outcome data.

### 2.4. Data Extraction and Synthesis

**Figure 1:**
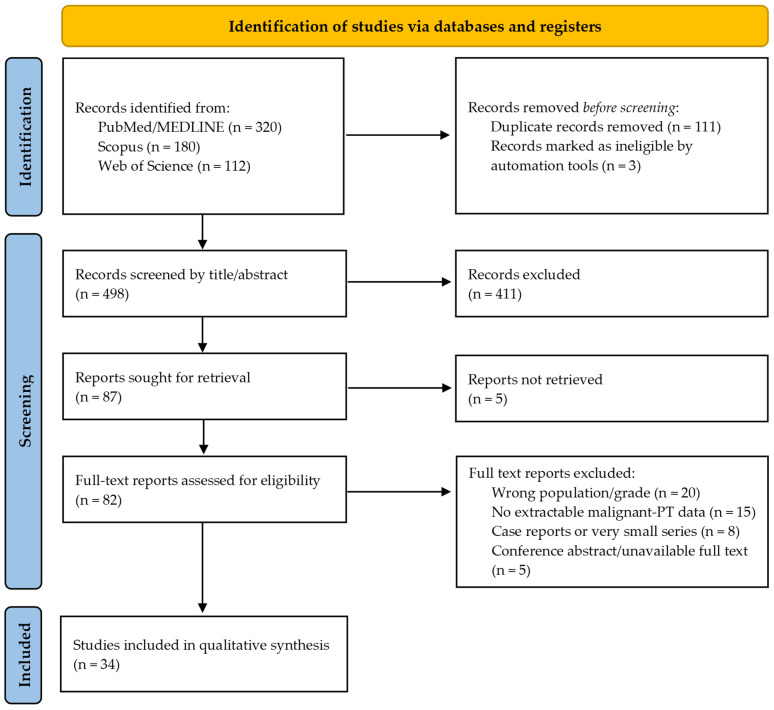
PRISMA 2020 flow diagram for study selection.

Two investigators independently screened titles and abstracts for eligibility. Full texts of potentially relevant studies were then reviewed. Discrepancies were resolved through discussion among the authors. The study selection process was summarised in a PRISMA 2020 flow diagram (Figure 1).

The following data were extracted using a standardised form: first author; year; country; study design; sample size; histologic grade; surgical approach; margin status/width; use of re-excision; RT details; follow-up duration; recurrence outcomes; survival outcomes; molecular alterations; immunohistochemical markers; and main conclusions.

Due to clinical, pathological, and methodological heterogeneity in study design and reporting, results were synthesised qualitatively rather than through formal meta-analysis. The primary synthesis was narrative and structured by three thematic domains: (i) molecular pathology, (ii) surgical margins and recurrence, and (iii) adjuvant RT.

The clinical case presented in this manuscript was integrated as an illustrative study highlighting the application of current evidence in clinical practice.

### 2.5. Methodological Limitations

A formal quantitative risk-of-bias scoring system was not used because of substantial heterogeneity in study design, treatment strategies, pathological reporting, and outcome definitions across studies. However, methodological limitations were assessed qualitatively according to study design, with attention to patient selection, sample size, comparability of groups, follow-up duration, outcome definition, and confounding by indication. These limitations were considered when interpreting the strength of evidence.

Consequently, the findings should be interpreted cautiously and viewed as hypothesis-generating rather than definitive.

To minimise overinterpretation, treatment-related conclusions in the present review were derived preferentially from retrospective cohort studies, multicentre analyses, registry-based studies, and systematic reviews/meta-analyses, whereas case reports and narrative reviews were retained only for contextual or illustrative purposes.

## 3. Results of the Systematic Review

### 3.1. Study Selection

A total of 612 records were identified through database searching. After removal of duplicates and screening, 34 studies met the eligibility criteria and were included in the qualitative synthesis. Among the selected records, only original cohort studies, registry-based analyses, multicentre studies, molecular studies, and immunohistochemical studies with original data were considered part of the primary core evidence synthesis. Systematic reviews/meta-analyses, narrative reviews and guidelines were used as contextual/background references, whereas single-case reports and very small case series were retained only as illustrative evidence. Therefore, case reports were not used to support conclusions regarding treatment efficacy, recurrence risk, survival outcomes, or prognostic molecular markers.

The PRISMA flowchart of the study selection is provided in Figure 1. Table 1, Table 2 and Table 3 summarise representative retrospective cohorts, multicentre studies, registry-based analyses, and molecular studies with original data included in the primary core evidence synthesis, whereas systematic reviews/meta-analyses were used only as supportive contextual references. The complete list of the 34 included studies is provided in the Supplementary Materials.

### 3.2. Surgical Margins and Recurrence

Large retrospective cohorts and population-based analyses indicate that malignant PTs account for approximately 10–20% of all phyllodes tumours and are associated with a clinically relevant risk of local recurrence and distant metastatic relapse during follow-up, most commonly involving the lung, bone, and brain [7,18,19,20,21,22,23,24]. The reported overall survival rates vary widely across series, reflecting differences in tumour stage, histological classification, treatment strategy, and follow-up duration [5,8,19,25,26,27]. Several studies consistently identify high mitotic activity, stromal overgrowth, infiltrative margins, and inadequate surgical margins as major predictors of recurrence and poorer outcomes. Additional retrospective studies have further confirmed the prognostic relevance of surgical margins, tumour grade, stromal overgrowth, and mitotic activity in predicting recurrence-free survival and long-term outcomes in PTs [26,27]. Therefore, estimates of metastatic risk should be interpreted cautiously rather than as fixed population-level rates.

Histological features that distinguish malignant PTs from borderline and/or benign lesions include stromal cellularity, mitotic activity, stromal overgrowth, and the nature of the tumour border [1,5]. Malignant PTs are characterised by hypercellularity, nuclear atypia, stromal overgrowth, increased mitotic activity (>10 mitoses per HPF) and an infiltrative border [6,8]. In the present case, despite the relatively small tumour size (1.7 cm on pathology), the patient’s lesion exhibited high-risk histological features, including marked stromal hypercellularity, nuclear atypia, increased mitotic activity (20/10 HPF), and focal infiltrative margins. These findings place the patient within a higher-risk subgroup described in a recent comparative series, highlighting that tumour biology rather than size alone should guide management decisions.

Diagnostic imaging lacks specificity for PTs, and preoperative biopsy often yields ambiguous results, making definitive diagnosis challenging. Because of the presence of both epithelial and stromal components, the differential diagnosis from fibroadenomas can be challenging. In a significant percentage of cases (up to 70%) [18], breast lesions that are initially presumed to be fibroadenomas and surgically excised due to increased size are ultimately diagnosed as PTs upon definitive histological analysis [18].

The typical mammographic appearance of all PTs is a well-circumscribed, hyperdense or isodense, round or oval mass [1,8]. Calcifications are usually absent, and the lesions are typically large and may exhibit rapid growth [1,5]. On ultrasound, PTs appear as solid, circumscribed masses with heterogeneous echogenicity [5]. Malignant PTs are more likely to present with irregular shapes, sizes greater than 7 cm, and cystic spaces [5,8]. On Magnetic Resonance Imaging (MRI), PTs typically present as well-circumscribed, round or oval masses that are isointense on T1-weighted images and hyperintense on T2-weighted images. In contrast to fibroadenomas, PTs are generally characterised by rapid contrast enhancement and washout kinetics, reflecting their hypervascularity and aggressive growth pattern [18].

Surgical excision with tumour-free margins is the standard treatment for all PTs [18,19,20,21]. Generally, it is not necessary to proceed with mastectomy [13], and breast-conserving surgery (BCS) is sufficient when negative margins can be achieved [8]. The impact of surgical margin width on local recurrence in malignant PTs remains a subject of debate (Table 1).

The available evidence consistently indicates that positive margins are associated with a higher risk of local recurrence. Wide local excision with negative margins remains the cornerstone of treatment. However, the necessity of an arbitrary 1 cm margin threshold is increasingly questioned. While the National Comprehensive Cancer Network (NCCN) guidelines traditionally recommend excision margins of at least 1 cm in breast-conserving surgery [1,22,23], a systematic review and meta-analysis of PTs’ surgical management suggested that positive margins were significantly associated with local recurrence, whereas margins <1 cm versus ≥1 cm were not significantly different overall [9]. Notably, Neron et al., in a multicentre retrospective study involving 212 patients with non-metastatic malignant PTs, suggested that margins of at least 3 mm may be sufficient when a genuinely negative margin is achieved, provided complete excision was achieved [24]. In clinical practice, histology and biology remain important even after technically adequate surgery due to the local recurrence patterns that are still observed despite margin-negative outcomes [28]. For malignant PTs with high-risk pathological features, re-excision is frequently recommended when margins are close, even when the tumour size is small. Nevertheless, other studies continue to suggest increased recurrence rates with close or positive margins [20,21].

Mastectomy may be required when adequate excision cannot be achieved, particularly in cases involving giant PTs [8]. When either mastectomy or even BCS results in large defects due to the size of the tumour, surgical planning must address not only oncologically adequate resection but also the need for an appropriate oncoplastic reconstruction. Such procedures can be technically challenging, especially when dealing with extensive defects. Several reconstructive approaches have been described in individual case reports and technical descriptions, including nipple-sparing mastectomy [29], autologous tissue breast reconstruction with latissimus dorsi (LD) flap, transverse rectus abdominis myocutaneous (TRAM) flap, or deep inferior epigastric perforator (DIEP) flap [29], retromuscular mammary implants with inferior dermal flap [30], and wide tumour resection with oncoplastic mastopexy [31]. However, these reports should be interpreted as illustrative surgical experiences rather than evidence supporting the superiority of one reconstructive strategy over another.

**Table 1:** Core studies on surgical margins and recurrence of malignant PTs.

Ref.	Author, Year	Study Design	Population	Margin Variable	Clinical Relevance
[9]	Wei et al., 2022	Systematic review/meta-analysis	34 studies across PTs grades	Positive vs. Negative; <1 cm vs. ≥1 cm	Positive margins increased LR; <1 cm vs. ≥1 cm not significantly different overall
[24]	Neron et al., 2020	Multicentre retrospective	Malignant PTs only	0–2 mm vs. ≥3 mm	Suggested ≥3 mm may be sufficient; no clear RT/CT effect in this cohort
[26]	Barrio et al., 2007	Retrospective	293 PTs	Margin status, histologic features	Margin status and malignant histology associated with increased recurrence risk and worse long-term outcomes
[28]	Choi et al., 2019	Retrospective	Borderline and malignant PTs after BCS	Margin-negative BCS cohort	Local recurrence patterns still observed despite margin-negative surgery

PTs predominantly spread via the haematogenous route, and axillary lymph node dissection is not routinely indicated [5,8,18,32]. However, if clinically or radiologically suspicious nodes are present [18] or if the PT is large [33], many authors recommend sentinel lymph node biopsy [3,33]. If metastatic involvement is detected, axillary dissection may be necessary [18]. Taken together, the available literature suggests a cautious and individualised approach to surgical management. The primary goal remains complete excision with histologically negative margins, while the clinical relevance of absolute margin width should be interpreted in relation to tumour grade, anatomical constraints, re-excision feasibility, and multidisciplinary discussion.

Reported local recurrence rates for malignant PTs range widely, from 12% to 65%, while distant metastasis rates vary between 16.7% and 30% across series [1,5,8,18,32]. Current NCCN guidelines recommend a follow-up period of at least three years [5], although several authors advocate for extended surveillance of up to five years due to the risk of late recurrence [32].

### 3.3. Adjuvant Radiotherapy

The role of adjuvant RT in malignant PTs is another area of ongoing debate [29,34]. Evidence for adjuvant RT remains retrospective and heterogeneous [18,35]. Institutional and multicentre studies suggest that RT may improve local control in selected patients with malignant PTs, especially in the presence of large tumour size, recurrent disease, borderline/positive or close margins, or breast-conserving treatment in higher-risk tumours. Nevertheless, RT is still employed in some clinical contexts (in 15.4–16.7% of cases) [20,36]. Some retrospective studies have reported lower local recurrence rates among patients receiving adjuvant RT, although the magnitude of benefit appears to vary according to margin status, tumour size, histological risk factors, and treatment selection [18,37]. Its use is therefore considered in cases with high-risk features. Wong et al. and Kim et al. [33,37] reported improved local control with adjuvant RT independent of margin status, while Gutnik et al. [20] emphasised the sarcoma-like biological behaviour of malignant PTs, supporting treatment strategies that differ from conventional breast carcinoma paradigms [35]. This effect may be particularly useful where local additional recurrence would lead to significant morbidity, such as chest wall recurrence following mastectomy [23], or in patients with negative prognostic factors [21,35,38]. Similarly, Belkacémi et al. reported improved local control rates in selected patients treated with adjuvant RT, but a definitive survival advantage could not be established because of the retrospective design and heterogeneity of the analysed cohorts [25]. However, database studies have not shown a consistent overall-survival benefit, and treatment allocation is vulnerable to confounding by indication because RT is preferentially used in patients with more adverse clinicopathologic features.

Currently, there is no indication for systemic chemotherapy in the management of localised PT cases [39]. However, some authors recommend systemic chemotherapy in cases of distant metastasis, following the NCCN guidelines for soft tissue sarcomas [8,18].

Overall, the available evidence suggests that adjuvant RT may improve local control in selected high-risk malignant PTs, particularly in patients with large tumours, recurrent disease, close or positive margins, or breast-conserving treatments. However, these findings derive mainly from retrospective studies and registry-based analyses, and no consistent overall survival benefit has been demonstrated.

### 3.4. Molecular Pathology and Prognostic Markers

Molecular studies have substantially advanced the biological understanding of PTs. *MED12* mutations represent one of the most frequently reported genetic alterations and are thought to occur early in tumorigenesis [12,14]. These mutations are also present in fibroepithelial lesions, supporting a shared pathway between fibroadenoma and PTs. In contrast, *TERT*-promoter mutations are enriched in PTs compared with fibroadenomas and appear to cooperate with *MED12* alterations in tumorigenesis [12,13,14]. Indeed, *TERT* promoter mutations have been associated with malignant transformation and tumour progression through activation of telomerase activity. With increasing PTs grade, genomic complexity rises, with more frequent *TP53*, *CDKN2A/B*, *NF1*, *SETD2*, *RB1*, *PIK3CA*, *PTEN*, and copy-number abnormalities observed in malignant or metastatic diseases [14,15]. These changes are associated with increasing genomic instability and aggressive tumour behaviour (Table 2).

These data support a progression model in which low-grade lesions are driven by early *MED12*-centred biology, whereas aggressive tumours acquire telomere-maintenance, cell-cycle, tumour-suppressor, and chromatin-remodelling alterations. This framework is highly relevant for differential diagnosis, especially in challenging spindle-cell lesions or unusual metastatic presentations and could eventually inform biomarker-driven stratification.

Immunohistochemical markers are less specific than genomic alterations but provide useful prognostic information. A higher Ki-67 proliferation index and stronger p53 expression have been associated with higher-grade PTs and worse outcomes, whereas stromal expression of CD34, CD99, and Bcl-2 may contribute to phenotypic characterisation but are insufficient as standalone prognostic tools [40,41] (Table 2).

**Table 2:** Core molecular and immunohistochemical PT studies.

Ref.	Author, Year	Study Type	Markers	Key Finding	Clinical Relevance
[12]	Yoshida et al., 2015	Molecular study	*TERT*-promoter, *MED12*	*TERT*-promoter mutations common and associated with *MED12*	Supports PTs’ biology distinct from fibroadenoma
[13]	Piscuoglio et al., 2016	Sequencing study	*TERT* alterations, actionable mutations	*TERT* alterations likely drive progression	Supports progression model and translational relevance
[14]	Garcia-Dios et al., 2018	Molecular study	*MED12*, *TERT*, *RBM15*	*MED12* heterogeneous; *TERT* more stable; *RBM15* in borderline/malignant PTs	Supports clonal evolution and recurrence biology
[15]	Tsang et al., 2022	Molecular profiling	*SETD2*, *TP53, CDKN2A/B*, *EGFR, PIK3CA*	Higher-grade PTs showed greater genomic complexity	Supports molecular escalation with increasing grade
[40]	Niezabitowski et al., 2001	Molecular study	*Ki-67*, *p53*	*Ki67* and *p53* associated with prognostic features	Practical prognostic marker in routine pathology
[41]	Ali et al., 2020	IHC study	*Ki-67*, *p53*	Higher expression associated with malignant PTs features	Supports IHC as adjunct prognostic information

**Table 3:** Representative retrospective cohort and multicentre studies on malignant PTs included in the primary core evidence synthesis.

Ref.	Author (Year)	Study Design	Malignant PT Cases (n)	Surgical Treatment	Adjuvant RT	Local Recurrence	Distant Metastasis	Main Findings
[7]	Ben Hassouna et al., 2006	Retrospective cohort	40	WLE/ Mastectomy	12%	23%	12%	Surgical margin status was the strongest predictor of local recurrence
[19]	Mituś et al., 2014	Retrospective	37	WLE/ Mastectomy	19%	21%	27%	RT reduced local recurrence but showed no overall survival benefit
[20]	Gutnik et al., 2022	Comparative analysis	82	WLE/ Mastectomy	15%	22%	16%	Malignant PTs showed sarcoma-like behaviour; the authors support sarcoma-oriented treatment
[24]	Neron et al., 2020	Multicentre retrospective	177	WLE/ Mastectomy	38%	19%	13%	Margins < 1 cm were acceptable; adjuvant RT improved local control
[25]	Belkacémi et al., 2008	Multicentre retrospective	443	WLE/ Mastectomy	18%	12%	8%	Adjuvant RT improved local control in selected high-risk patients; no definitive survival benefit demonstrated
[27]	Ji et al., 2022	Retrospective cohort	404	WLE/ Mastectomy	—	14%	—	Margin status and tumour grade associated with recurrence and prognosis
[35]	Oladeru et al. (2020)	SEER-based retrospective	1353 *	WLE/ Mastectomy	16.7%	—	—	RT use associated with improved local control in high-risk tumours
[36]	Wong et al. (2020)	Retrospective	108	WLE/ Mastectomy	29%	18%	10%	Adjuvant RT improved local control regardless of margin status
[37]	Kim et al. (2017)	SEER database analysis	478	WLE/ Mastectomy	15.4%	—	—	RT reduced local recurrence but did not improve overall survival
[38]	Liu et al. (2023)	Real-world retrospective	188	WLE/ Mastectomy	21%	24%	18%	Large tumour size and malignant histology correlated with worse outcomes

* Includes malignant and high-risk PTs analysed for RT patterns.

However, although molecular profiling has improved the biological understanding of malignant PTs, the prognostic and therapeutic utility of these markers remains investigational. At present, *MED12*, *TERT promoter*, *TP53*, *CDKN2A/B*, *SETD2*, *RB1*, and related alterations should not be interpreted as validated standalone markers for treatment selection or prognostic stratification outside research or highly selected multidisciplinary contexts.

## 4. Case Presentation as Clinical Illustration

**Figure 2:**
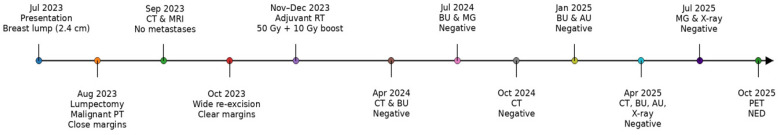
Clinical timeline of diagnosis, treatment, and follow-up.

A 44-year-old Italian woman presented to our IRCCS-CROB Hospital with a lump in her left breast in July 2023; the lump had a maximum diameter of 2.4 cm on radiological examination (Figure 2). She reported no pain or discomfort.

The patient’s family history was notable for a maternal great-grandmother with breast cancer and a maternal grandmother with hematologic malignancy. Her medical history included post-herpetic meningoencephalitis, a right humerus fracture, thyroiditis with hypothyroidism, and pre-menopausal status. She was a non-smoker, did not consume alcohol, and had three children.

A bilateral mammogram revealed a 2.4 cm circumscribed, dense round mass in the upper outer quadrant (UOQ) of the left breast, with regular margins and an absence of calcifications. At the bilateral breast ultrasound examination, it appeared as a solid, homogeneous, hypoechogenic mass, suggestive of fibroadenoma versus PT; no additional solid nodular lesions nor axillary lymphadenopathies were identified bilaterally. These findings raised a clinical suspicion of PT.

**Figure 3:**
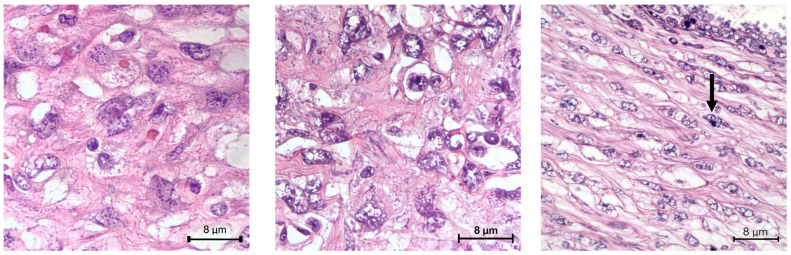
PT highly pleomorphic neoplastic cells with vesicular nuclei and atypical mitosis (arrow) (×40).

**Figure 4:**
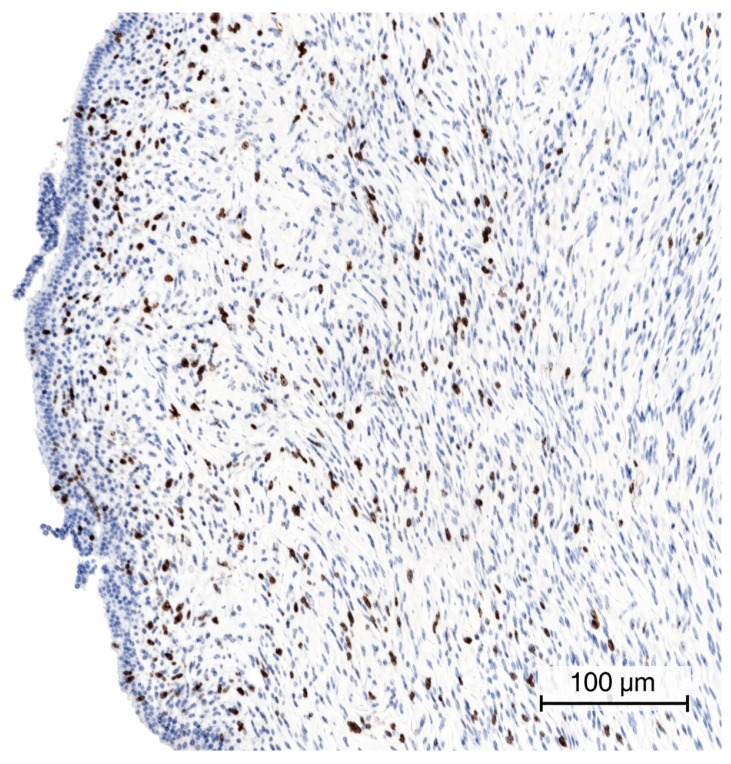
PT high mitotic index (Ki-67) intensified around the breast duct (×20).

The patient underwent a lumpectomy in August (Figure 2). The histopathological examination confirmed the nature of the lesion: a malignant PT measuring 1.7 cm in maximum diameter, with a marked stromal hypercellularity, nuclear atypia, mitotic index of up to 20/10 high power field (HPF) (Figure 3), and Ki-67 proliferation index of 20% (Figure 4). Histopathological evaluation showed tumour cells located within less than 1 mm of the surgical margin, which was therefore classified as a close margin.

Based on these findings, a total body Computed Tomography (CT) scan and an MRI of the dorsal and lumbar spine were performed for tumour staging. Both examinations showed no evidence of metastases. Given the malignant histology and the presence of a close surgical margin (<1 mm), the multidisciplinary team recommended wide local re-excision to achieve a clear margin and reduce the risk of local recurrence.

**Figure 5:**
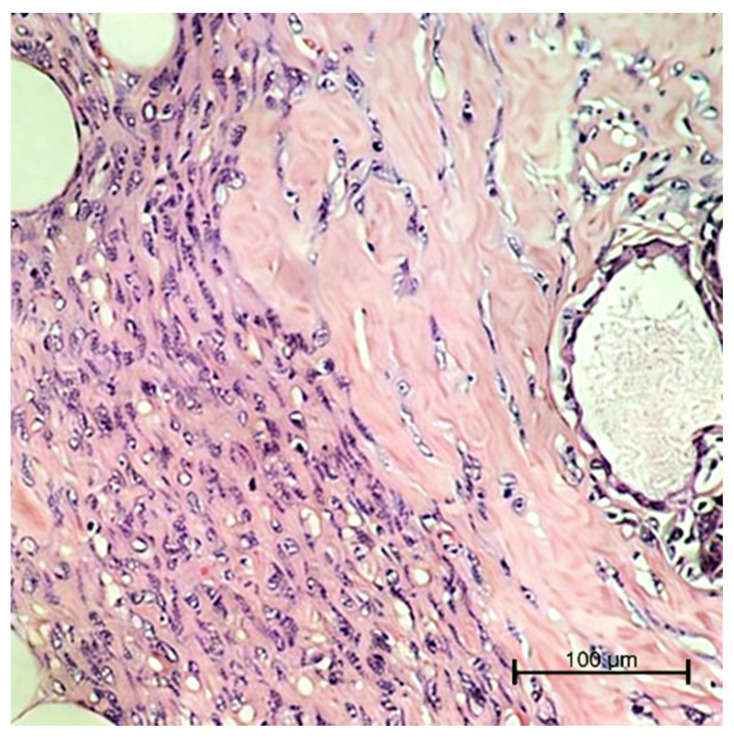
PT abrupt transition from hypocellular sclerotic breast tissue to a highly cellular neoplastic area (×20).

The histopathological examination confirmed that the breast parenchyma was the site of a predominantly well-circumscribed nodular formation with expansive margins, focally with an infiltrative growth pattern, consisting of a diffuse and richly cellular proliferation of stromal elements, some of which appeared highly atypical and pleomorphic (Figure 5).

**Figure 6:**
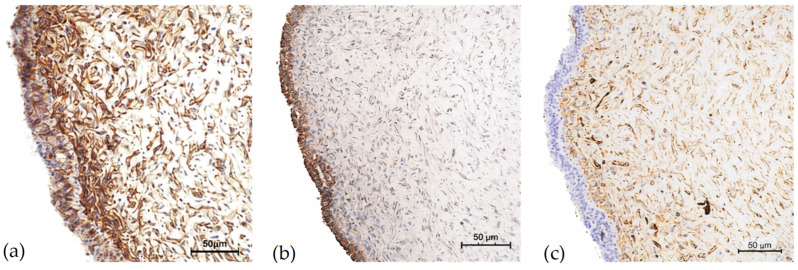
PT cells positive for CD99 (**a**), Bcl2 (**b**) and CD34 (**c**) (×20).

The neoplastic cells were also positive for CD99, Bcl2, and CD34 (Figure 6).

The combination of CT, MRI and histopathological findings confirmed the diagnosis of a malignant PT (according to the latest WHO classification, 2019 [2]) and excluded the presence of additional neoplastic disease.

To carefully evaluate the different available treatment options, the breast surgeon presented the patient’s clinical case to a multidisciplinary oncology team that included breast surgeons, oncologists, and radiation oncologists. Given the malignant histology, high mitotic rate, and initially close margins, the multidisciplinary team proposed postoperative complementary RT as part of the clinical treatment strategy.

**Figure 7:**
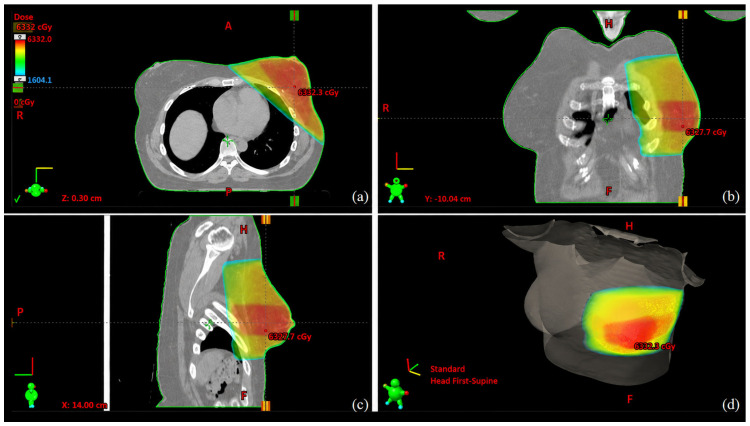
3D-CRT of the left breast patient with daily dosages of 2 Gy to a total dose of 50 Gy in 25 fractions: (**a**) transversal, (**b**) frontal, (**c**) sagittal, and (**d**) model view plan sums. Dashed lines indicate the orthogonal reference axes crossing at the treatment isocentre. Orientation labels correspond to patient anatomy and positioning: H = head, F = feet, R = right, L = left, A = anterior, and P = posterior. The coloured scale represents radiation dose distribution (cGy). The orientation icon indicates the patient setup in the head-first supine position.

While hypofractionated RT is standard in breast-conserving therapy, evidence supporting its use in malignant PTs is limited. Therefore, a conventional sarcoma-based regimen was selected (Figure 2) [22,23]. The patient underwent adjuvant whole breast 3D conformal radiation therapy (3D-CRT) consisting of with twenty-five daily fraction dosages of 2.0 Gray (Gy) up to a total dose of 50 Gy over 5 weeks (November–December 2023), followed by a 10 Gy boost in five fractions on the tumour bed and surgical scar using photons and the Intensity Modulated Radiation Therapy (IMRT) technique, for a cumulative total dose of 60 Gy (Figure 7). Neither acute nor subacute toxicities were recorded.

Follow-up evaluations were performed at 3, 6, and 12 months in 2024 and at 18 and 24 months in 2025. Imaging studies, including breast ultrasound (BU) and abdominal ultrasound (AU), total-body CT, mammography (MG), and total-body Positron Emission Tomography (PET), together with blood tests for tumour markers (CEA, CA-125, CA-15.3, CA-19.9), showed no evidence of disease (NED) at all follow-up time points (Figure 2). Although the patient remains disease-free at the latest follow-up, long-term surveillance is planned given the potential for late recurrence in malignant PTs.

The patient provided written informed consent for publication of the case and accompanying images.

## 5. Integrated Discussion

PTs of the breast are biphasic neoplasms composed of both an epithelial and a stromal cellular component, a feature essential for diagnosis [18]. The presence of these two components is crucial for diagnosis. The neoplastic component is identified by the stromal fronds, while the epithelial component is benign and organised in a bilayer that covers either tubular or leaf-like structures [18].

Malignant PTs of the breast remain rare entities, with an average annual age-adjusted incidence of 2.1 cases per one million women [19], and most available evidence derives from retrospective series and institutional experiences rather than prospective trials. While their general epidemiology and histopathological features are well established, significant uncertainties persist regarding optimal surgical margins, the role of re-excision for close margins, and the indication for adjuvant RT, particularly in small malignant tumours such as the one presented here. The present PRISMA-guided qualitative systematic review suggests that surgical margin status remains one of the most consistently reported factors associated with local recurrence [20,21,22,23,24,25,26,27,28]. Large retrospective cohorts and institutional series, including studies by Barrio et al. and Ji et al., further support the association between margin status, stromal overgrowth, malignant histology, and recurrence risk, although substantial heterogeneity remains across published studies [26,27].

Although traditional guidelines recommend margins of at least 1 cm [22,23], emerging evidence suggests that smaller margins may be acceptable when complete excision is achieved. However, re-excision is often recommended in cases of malignant histology with close margins, particularly when additional risk factors are present. In this context, the present clinical illustration reflects a cautious multidisciplinary approach. Despite the relatively small tumour size, the lesion exhibited several adverse pathological features, including stromal hypercellularity, marked atypia, infiltrative growth pattern, and high mitotic activity. These findings influenced the decision to perform re-excision after the initial lumpectomy in order to obtain clearer margins and potentially reduce recurrence risk. This case therefore reflects real-world clinical decision-making in an area where evidence remains conflicting.

The role of adjuvant RT in malignant PTs also remains controversial. Available retrospective institutional and registry-based studies suggest a potential reduction in local recurrence among selected high-risk patients treated with adjuvant RT [32,33,34,35,36,37,38]; however, these findings remain limited by retrospective design, heterogeneity in patient selection, and confounding by indication. This is particularly relevant in patients with large tumours, high mitotic activity, or close surgical margins. These findings contextualise the decision in the case presented as a clinical illustration, where the decision to administer postoperative RT was influenced not by tumour size alone, but by the combination of malignant histology, high mitotic activity, and initially close margins. Indeed, adjuvant RT (60 Gy total dose) was administered to the patient following a multidisciplinary discussion that selected conventional fractionation because the stromal component of malignant PTs shares biological similarities with soft tissue sarcomas. These treatment principles and the absence of validated hypofractionated protocols for PTs aim to reduce recurrence risk given the malignant histology and initial close margins. Importantly, although hypofractionated RT is now standard in breast-conserving treatment for invasive breast carcinoma, evidence supporting its use in malignant PTs is lacking. Given the sarcoma-like biological behaviour of the stromal component, a conventional fractionation regimen was selected, consistent with approaches reported in sarcoma-oriented series and contemporary clinical practice surveys [32,35]. This case therefore contributes to the limited clinical documentation of fractionation choices in PTs and highlights another area where prospective data are needed.

Molecular studies have substantially improved the biological understanding of PTs. Alterations involving *MED12* and *TERT* promoter mutations appear to play important roles in tumorigenesis and progression, while higher-grade tumours frequently demonstrate increasing genomic complexity involving *TP53*, *CDKN2A/B*, *SETD2*, *RB1*, and *PI3K*-pathway alterations. However, although these findings are biologically promising, their routine clinical application for prognostic stratification or therapeutic decision-making remains investigational and has not yet been validated prospectively.

Overall, although the tumour in this case was small and the clinical presentation of this case may appear typical, its educational value lies in illustrating how multiple borderline or controversial management decisions are addressed in practice: re-excision for close margins, the real-world application of multidisciplinary decision-making, selective use of adjuvant RT, and choice of radiation fractionation in the absence of high-level evidence. Therefore, the present case illustrates how multidisciplinary evaluation can guide individualised management strategies. This individualised, multidisciplinary approach mirrors contemporary practice patterns reported in international surveys, underscoring the absence of universally accepted treatment algorithms.

The accompanying structured review summarised in Table 3 further contextualises these decisions within the current literature and emphasises the persistent gaps in evidence that justify continued reporting and analysis of such cases.

A limitation of the present report is the relatively short follow-up period, which currently extends to 24 months. Although the patient remains disease-free, malignant PTs are characterised by a potential risk of late local recurrence and distant metastasis. Therefore, a longer follow-up is required before definitive conclusions regarding long-term disease control can be drawn. In accordance with recommendations reported in retrospective series and available guideline-based follow-up strategies, continued surveillance has been planned for this patient, including periodic clinical examination and breast imaging for at least five years to detect possible late recurrences.

Several limitations must be acknowledged. The rarity of malignant PTs represents a major limitation for evidence generation. Most available studies are retrospective, involve relatively small populations, and frequently differ in pathological classification, treatment strategies, follow-up duration, and outcome reporting. Moreover, molecular markers remain promising but investigational, and their clinical utility requires validation in larger prospective cohorts. Consequently, the findings of the present review should be interpreted as a qualitative evidence synthesis rather than definitive treatment guidance.

Despite these limitations, this study has several strengths, including the integration of real-world multidisciplinary clinical decision-making in a rare tumour setting with a structured qualitative synthesis of available evidence on clinically relevant domains (e.g., surgical margins, adjuvant RT, and molecular pathology).

## 6. Clinical Implications and Future Directions

Because malignant PTs are rare, progress will depend on multicentre registries and tissue-linked outcome databases that combine standardised pathology review, centralised genomic profiling, RT details, and longitudinal follow-up. The integration of molecular profiling with clinical and histopathological factors may improve risk stratification in patients with PTs. Future research should focus on identifying biomarkers capable of predicting recurrence and metastatic potential.

Next-generation sequencing approaches may provide insights into targeted therapeutic strategies, particularly for advanced or metastatic disease.

Another important challenge in the management of malignant PTs is the absence of standardised long-term follow-up protocols. Recurrences may occur several years after initial treatment, and current surveillance strategies are largely based on institutional practices rather than prospective evidence.

Large multicentre registries and prospective observational studies would be essential to better define optimal surgical margins, clarify the role of adjuvant RT, evaluate the prognostic significance of molecular alterations, and establish the optimal frequency and duration of imaging and clinical follow-up.

## 7. Conclusions

Malignant PTs of the breast are rare fibroepithelial neoplasms characterised by a biphasic cellular component that, in only a small percentage of cases, show malignant aspects. Owing to the rarity of malignant PTs and the predominance of retrospective evidence, current management strategies should be interpreted cautiously and not as definitive evidence-based treatment algorithms.

The present PRISMA-guided qualitative systematic review supports that complete surgical excision with histologically negative margins remains the cornerstone of treatment associated with improved local control.

Available retrospective studies suggest that adjuvant RT may improve local control in selected high-risk patients, particularly in the presence of malignant histology, large tumour size, recurrent disease, or close/positive surgical margins. However, these findings remain limited by retrospective study design, treatment-selection bias, heterogeneity across published series, and the absence of prospective randomised evidence demonstrating a consistent survival benefit.

Emerging molecular profiling has also improved the biological understanding of PTs. Recurrent alterations involving *MED12*, the *TERT*-promoter, *TP53*, *NF1*, *CDKN2A/B*, *SETD2*, and related pathways support a model of stepwise biological progression and offer a framework for improved diagnosis, prognosis, and future therapeutic exploration. Nevertheless, the clinical utility of currently available molecular biomarkers remains investigational and requires validation in larger prospective studies. Therefore, treatment decisions should continue to be individualised within a multidisciplinary setting until higher-quality prospective evidence becomes available.

The clinical case presented highlights the importance of complete surgical excision with adequate margins followed by adjuvant RT, which achieved short-term disease control and resulted in a disease-free status at 24 months. This case reinforces the importance of a multidisciplinary evaluation and individualised treatment strategies in the presence of adverse pathological features and contributes a structured synthesis of current evidence to support clinical decision-making; however, this observation should be interpreted within the limitations inherent to single-case experience.

Overall, future prospective multicentre and collaborative studies integrating genomic profiling with clinical outcomes are needed to clarify the role of RT in this rare pathology, optimise surgical strategies, develop personalised therapeutic approaches, define evidence-based management strategies and long-term follow-up protocols, and validate molecular prognostic markers to improve patient outcomes.

## Data Availability

The original contributions presented in this study are included in the article. Further inquiries can be directed to the corresponding author.

## Supplementary Materials

The following supporting information can be downloaded at: https://www.mdpi.com/article/10.3390/ijms27104376/s1.
